# Observation of negative differential resistance in mesoscopic graphene oxide devices

**DOI:** 10.1038/s41598-018-22355-0

**Published:** 2018-05-08

**Authors:** Servin Rathi, Inyeal Lee, Moonshik Kang, Dongsuk Lim, Yoontae Lee, Serhan Yamacli, Han-Ik Joh, Seongsu Kim, Sang-Woo Kim, Sun Jin Yun, Sukwon Choi, Gil-Ho Kim

**Affiliations:** 1 Samsung-SKKU Graphene Center, Sungkyunkwan Advanced Institute of Nanotechnology (SAINT) and School of Electronics and Electrical Engineering, Sungkyunkwan University, Suwon, 16419 Korea; 2 Manufacturing Engineering Team, Memory Division, Samsung Electronics Co, Hwasung, 18396 Korea; 3 Department of Electrical-Electronics Engineering, Nuh Naci Yazgan University, 38090 Kayseri, Turkey; 4 Department of Energy Engineering, Konkuk University, 120 Neungdong-ro, Gwangjin-gu, Seoul, 05029 South Korea; 5 School of Advanced Materials Science and Engineering, SKKU Advanced Institute of Nanotechnology (SAINT), Center for Human Interface Nanotechnology (HINT), and IBS Center for Integrated Nanostructure Physics, Sungkyunkwan University, Suwon, 16419 Korea; 6 ICT Components and Materials Technology Research Division, Electronics and Telecommunications Research Institute, Daejeon, 34129 Korea; 7 Department of Mechanical and Nuclear Engineering, The Pennsylvania State University, University Park, PA, 16802 USA

## Abstract

The fractions of various functional groups in graphene oxide (GO) are directly related to its electrical and chemical properties and can be controlled by various reduction methods like thermal, chemical and optical. However, a method with sufficient controllability to regulate the reduction process has been missing. In this work, a hybrid method of thermal and joule heating processes is demonstrated where a progressive control of the ratio of various functional groups can be achieved in a localized area. With this precise control of carbon-oxygen ratio, negative differential resistance (NDR) is observed in the current-voltage characteristics of a two-terminal device in the ambient environment due to charge-activated electrochemical reactions at the GO surface. This experimental observation correlates with the optical and chemical characterizations. This NDR behavior offers new opportunities for the fabrication and application of such novel electronic devices in a wide range of devices applications including switches and oscillators.

## Introduction

In recent years, two-dimensional (2D) atomic thin materials including graphene, graphene oxide, and transition metal dichalcogenides (TMDs), have generated considerable interest for device applications owing to their versatility, scalability, and stability^1–5^. Various device structures such as vertical tunneling transistors, lateral chemical and field doped diodes, light emitting diodes, and photovoltaic cells have been demonstrated with different 2D materials^6–10^. Furthermore, negative differential resistance (NDR) has also been reported in 2D-material-based systems (such as graphene/h-BN/graphene) including TMD-based (e.g., molybdenum disulfide or tungsten diselenide) heterostructure devices^6,11–15^. NDR, one of the most interesting quantum electronic tunneling transport phenomena, has found applications in high-frequency oscillators, frequency multipliers, and logic gates^16,17^. Although historically observed in a degenerate doped solid-state Esaki diodes via inter-band tunneling, NDR has also been realized in double quantum well heterostructures through resonant and off-resonant tunneling with an improved peak-to-valley ratio (PVR)^18,19^. Several groups have also observed and simulated NDR in molecular systems such as oligophenylene ethynylene molecular junctions, single benzene rings and other organic systems including carbon nanotubes, graphene, and MoS_2_ based nanoribbons whereas it has been mostly studied via quantum simulation techniques^15,20–23^. The mechanism behind the NDR phenomenon in molecular systems is conceptually different from that observed in typical semiconductor material systems, and has been attributed to various factors including electric field induced alignment of molecular orbitals, charge transfer, coupling with contact electrode, and conformational variation in the molecule^20,21^. In molecular and mesoscopic systems, NDR is also found to be very sensitive to doping and strain, particularly in graphene and MoS_2_ nanoribbons where factors including edge doping, twisted channel layer, and edge states are critical parameters affecting the device performance^24–26^. Furthermore, for practical applications, the experimental observation of NDR in molecular and 2D-material-based heterostructure devices is still far away from scalable production, owing to the complicated transfer and fabrication techniques^7^. On the other hand, due to the requirement of atomic size precision and doping methods, the realization of NDR in nanoribbons has remained elusive^26^. In this work, graphene oxide (GO), a widely available and studied 2D material, is employed to realize workable and scalable NDR devices.

A GO suspension of a few atomic-thick oxidized graphene layers is usually synthesized using a modified Hummers’ method and can be easily assembled between the metal electrodes using various methods like drop and dried, dielectrophoresis, etc^27,28^. The study of the chemical and electrical properties of GO has shown that it behaves like a wide band gap insulator with a band gap in the range of ~0.7–2.7 eV with intermittent localized energy states due to the presence of various functional groups such as carboxyl, hydroxyl, and epoxy groups^29,30^. Any variation in the density of these functional groups in GO through various reduction routes–e.g., thermal, Joule heating, microwave, chemical, and optical methods–affects various properties including thermal and electrical conductivities, reflectance, chemical reactivity, mobility and concentration of charge carriers^31–33^. All reduction process of GO usually increases the carbon-to-oxygen ratio by healing and reconstructing defects in its hexagonal carbon frame, which also increases the electrical conductivity, thus making it feasible for conductive electronics^34–36^. On the other hand, the defects and residual functional groups in the reduced GO (rGO) work as active chemical sites and have been explored for various sensing applications^37^. Therefore, the reduction of GO presents a facile and low-cost path to obtain graphene, albeit with residual chemically active functional groups and an imperfect honeycomb lattice.

In this study, we have precisely optimized the GO reduction process by combining the use of thermal and Joule heating based reduction mechanisms where a mild preheating was followed by controlled Joule heating cycles until NDR was observed in partially reduced GO. The advantage of this two-step reduction scheme is that the reduction process can be controlled through Joule heating by injecting controlled current in the GO device, whereas the preheating thermal step facilitates establishing a relatively less resistive conduction path during the initial application of voltage bias. Here, we show that, through this method, one could, in principle, completely and continuously change the carbon-to-oxygen ratio in the GO device.

It has been observed in simulation results based on density functional theory (DFT) and non-equilibrium Green’s function (NEGF) that, besides other factors, doping along the edges in graphene nanoribbons (GNRs) is a critical parameter to initiate and modulate the NDR behavior^38^. On a similar analogy, we have demonstrated through simulations based on DFT and NEGF, that NDR can be observed in GO-based devices at a certain carbon-to-oxygen ratio along the conduction path, whereas the experimentally observed NDR is found to be associated with the electric charge induced electrochemical reactions at the GO interface in the ambient environment, which leads to p-type doping in GO layers resulting in the observation of NDR.

## Results and Discussion

**Figure 1:**
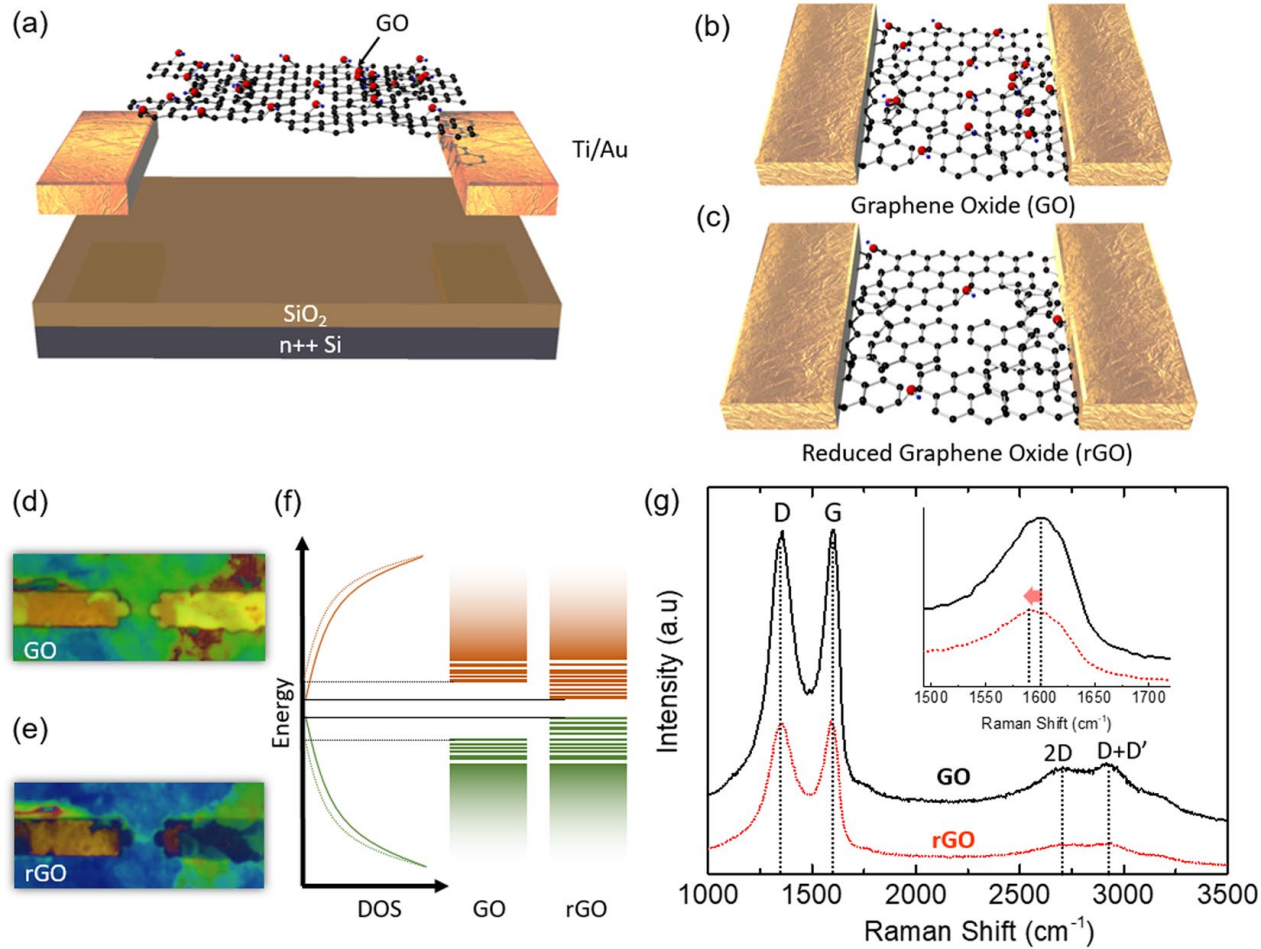
Illustration and optical images of fabricated devices, and Raman spectra. (**a**–**c**) Schematic of the fabricated devices. (**d**–**e**) Optical microscope image (the scale bar represents 4 µm) of the pre- and post-reduced device illustrating the color variation in the reduction process. (**f**) Schematic energy band diagram illustrating localized electronic states at the band edges; the energy gap between conduction and valence band edges depends on the molecular compositions and their configurations. (**g**) Raman spectra of GO and rGO. The inset shows the zoomed-in graph of the G peak.

Figure 1(a) shows the schematic of the tested device, in which the Ti/Au metal contacts were used to apply voltage bias across the GO layers. By applying a bias across the contacts, the amount of Joule heating was precisely controlled to alter the carbon-to-oxygen ratio in the GO flakes. As shown in Fig. 1(b), the untreated sample had a high density of various oxygen functional groups, such as carboxyl, hydroxyl, epoxy groups, etc., indicated by the red entities in the main channel area. The density of these functional groups can be controlled by various chemical, thermal, or Joule heating processes. In this study, a hybrid approach based on the combination of thermal and Joule heating was utilized to precisely control the density of these functional groups in the GO layers (see the Fig. 1(c) for reduced GO; rGO). The reduction of GO during the thermal reduction process, which is revealed by the color change (yellow to blue) in the optical images illustrated in Fig. 1(d–e), (see Supplementary Information, section A, for details regarding the thickness profile). The schematic band diagram of GO and rGO as illustrated in Fig. 1(f) shows a schematic band diagram of GO and rGO, where the lines represent the localized electronic states near the band gap edges, and their density and position depend on the type and density of defects in the GO structures. Although the healing of the GO structures by the thermal process resulted in the improvement of various electrical, optical, and sensing properties, but even after the reduction, the opened band gap cannot be completely recovered to that of graphene (no band gap state), owing to the high density of defects, residual traces of oxygen functional groups, and strong confinement effects in small sp^2^ domains. Additionally, no exact correlation was found between the band gap and degree of GO reduction, as the GO band gap varied over a wide range of ~0.7–2.7 eV. In order to study the effects of thermal reduction on the modification of various chemical bonding in the GO layers, detailed structural and chemical characterizations were conducted through Raman and X-ray photoelectron spectroscopy (XPS) (refer to “Characterization with Raman and XPS spectroscopies” section for details). Raman measurements were performed at room temperature with a 532 nm laser to obtain detailed information of the chemical and structural alterations induced by the thermal reduction. Fig. 1(g) show the Raman spectra with two remarkable peaks at 1340 and 1563 cm^−1^, i.e., the D and G peaks, which are related to the presence of defects and relative degree of graphitization, respectively, in GO layers. After thermal reduction (400 °C for 3 min), the G peak shifted from 1602 to 1593 cm^−1^, which indicated the graphitization of GO, confirming the reduction of the functional groups in the GO layers. This condition is illustrated in Fig. 1(c) where the decrease in density of red entities indicates the reducing effects of the thermal process. Furthermore, the prominence of the D peak in the Raman spectra, even after thermal reduction, revealed the existence of defects at the functionalized sites even after the removal of the oxygen functional groups. In addition to the D and G peaks, broad peaks could be observed in the high-frequency end of the spectrum, in the range of ~2690–2300 cm^−1^; these peaks are usually composed of D peak overtones and a combination of D and G peaks, named as 2D, D + G, and 2D′. (see Supplementary Information, Section B for detailed Raman measurement and comparison between thermal and Joule heating reduced GO). These peaks and bands, as observed in the GO Raman spectra, were consistent with previously published results^39,40^.

**Figure 2:**
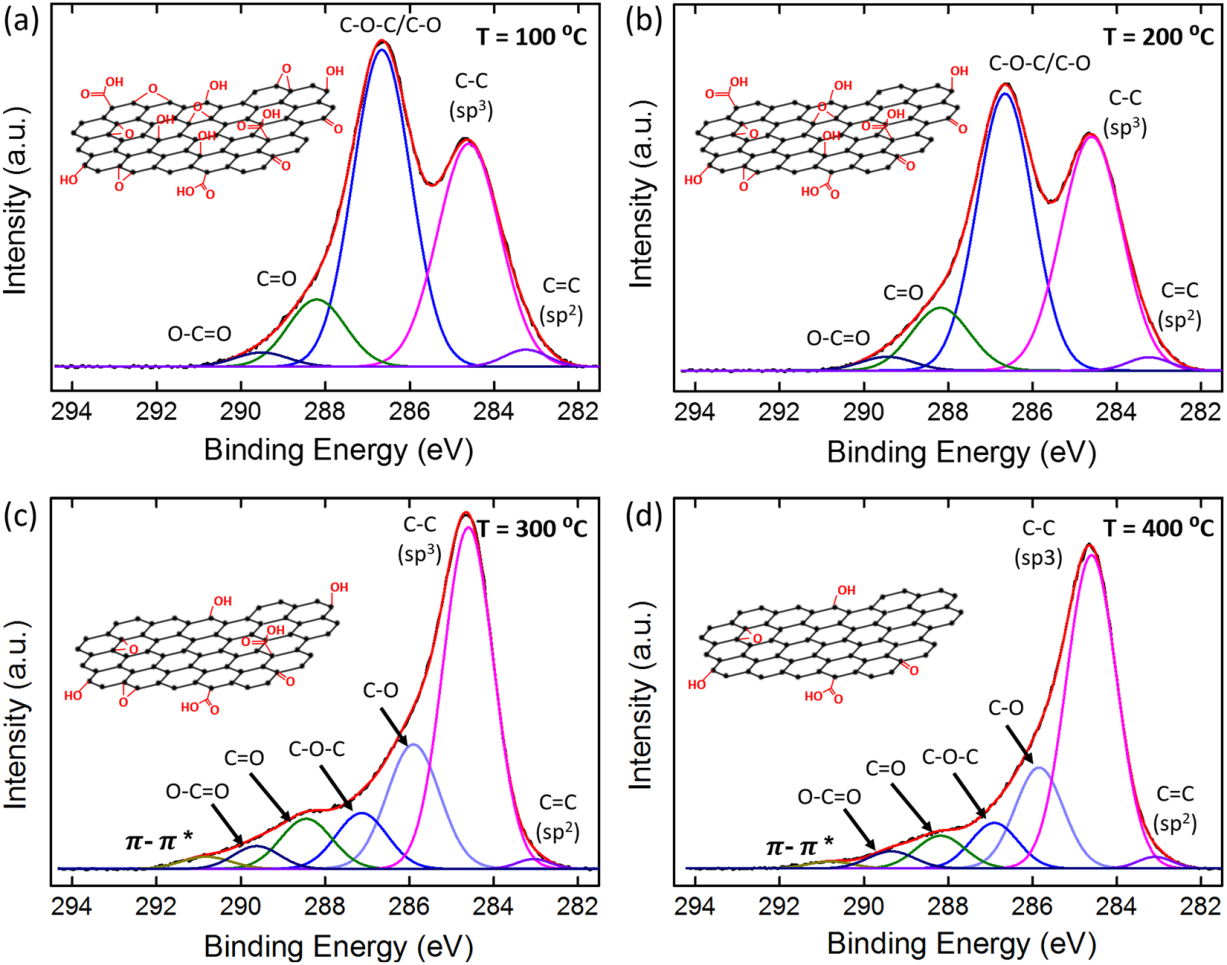
Chemical bonding analysis of graphene oxide GO. X-ray photoelectron spectroscopy peaks of carbon and oxygen functional group of GO flakes annealed at temperatures of (**a**) 100, (**b**) 200, (**c**) 300, and (**d**) 400 °C for 3 min.

For an in-depth analysis of chemical compositions of GO and rGO layers, XPS (ESCA 2000, VG Microtech, UK) measurement was conducted for the samples thermally annealed at 100, 200, 300, and 400 °C, respectively. Fig. 2 shows the deconvoluted C1s peaks after Gaussian-Lorentzian fitting and Shirley background corrections, obtained upon controlled thermal annealing at various temperatures. (see Supplementary Information, Section C for detailed measurement of O1s XPS spectra for GO and rGO) Various functional groups associated with GO could be inferred and identified by their peak position in the deconvoluted XPS spectra, like carbon (C–C sp^2^; 283.10 eV and sp^3^; 284.60 eV), oxygen-based hydroxyl (C–O; 285.85 eV), epoxy (C–O–C; 286. 95 eV), carbonyl (C = O; 288.25 eV), carboxylate (O–C = O; 289.40 eV), and pi-pi transition groups (π–π; 290.85 eV). These values were in good agreement with published results^27,28^. It may be noted that the C-O and C-O-C peaks were not convoluted in the samples annealed at 100 and 200 °C. By increasing the annealing temperature, the peaks related to these oxygen functional groups decreases in intensity, which also indicates an increase in carbon-to-oxygen ratio, thereby confirming the reduction of the GO layers. According to Fig. 2(a) and b, until 200 °C, the chemical composition, and functional groups exhibited only a marginal variation and an observable change was only seen in the intensity ratio of C–O, and C–C peaks, whereas other functional groups like C = O and O–C = O show negligible alteration. However, the XPS spectra observed at temperatures of 300 °C and above (Fig. 2(c,d)) show a major reduction in the peaks of almost all the functional group. Therefore, the temperature in the range of 100–200 °C can be considered as a safe window where major chemical variations do not occur in GO. Moreover, this annealing process under 200 °C can remove the residual water molecules in GO layers which may hinder in the current measurement and its analysis. This observation is very critical to this study, as, for our hybrid reduction process, which includes both thermal and Joule heating processes, we thermally treated the devices within this safe temperature window, and the critical and final carbon-to-oxygen ratio was achieved by the Joule heating process. The XPS analysis also confirm and agree with the Raman analysis, which showed the restoration of sp^2^ mesh or graphitization of GO with the blue shift of the G peak. The thermal annealing temperature directly affects the recovery of the sp^2^ honeycomb lattice by controlling the density of the functional groups which are represented by sp^3^ domains and clusters. Therefore, the thermal annealing can be utilized to tune various electrical, chemical, and sensing properties of the GO layers (see Supplementary Information, Section D for detailed measurement of electrical properties at various annealing temperature and GO concentration).

**Figure 3:**
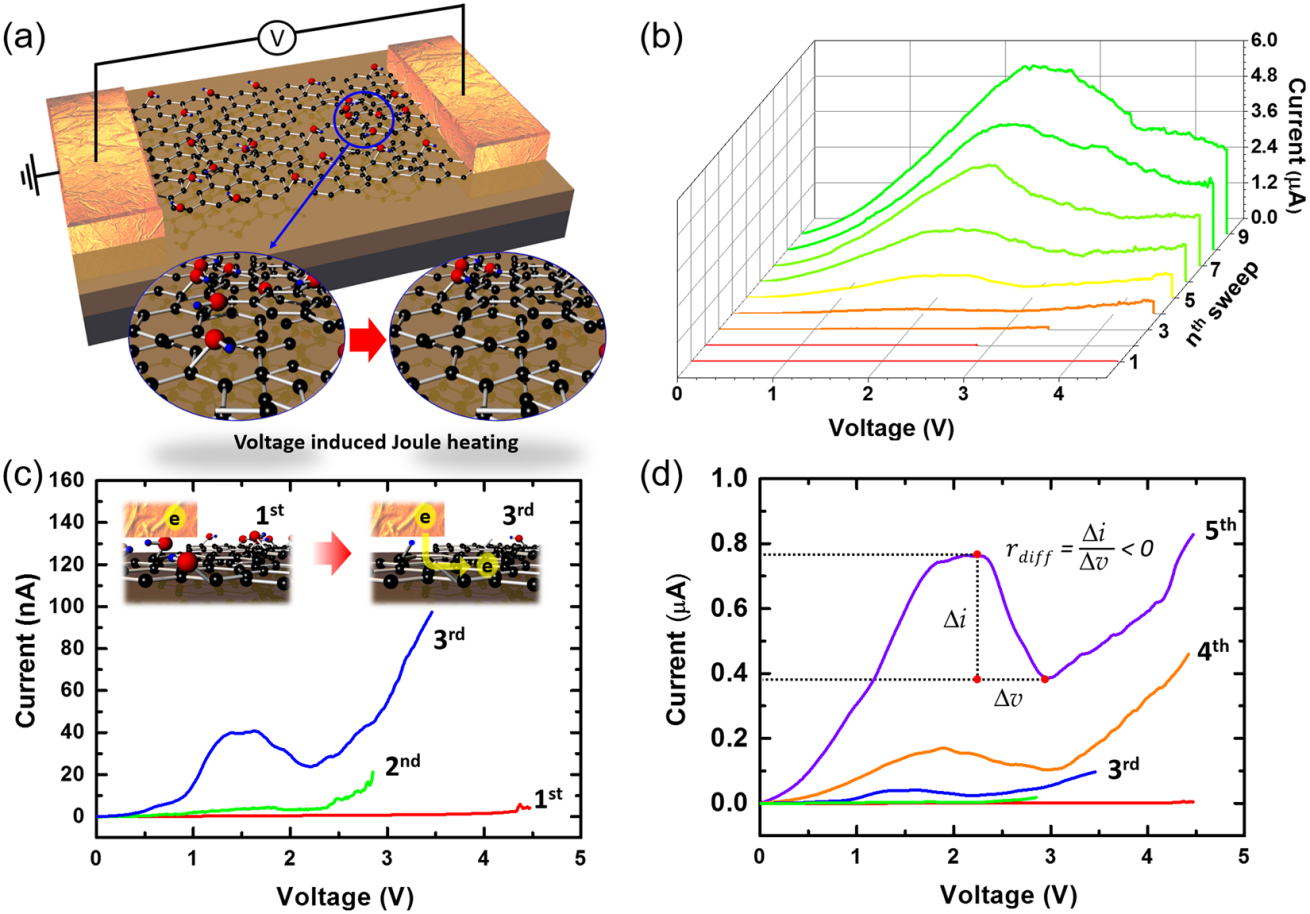
Observation of negative differential resistance (NDR) in reduced graphene oxide device. (**a**) Schematic illustration of the device under measurement, where the inset images show the removal of oxygen functional groups by Joule heating process. I–V characteristics for (**b**) successive voltage sweeps from 1^st^ to 9^th^, (**c**) selective voltage sweeps from 1^st^ to 3^rd^ with the inset illustrating the transformation of GO-metal contacts from resistive to conductive after Joule heating induced reduction of GO in the successive voltage sweeps. (**d**) selective voltage sweeps from 1^st^ to 5^th^ with the description of key NDR metric.

After studying the effect of the thermal process on the structural and chemical properties of the GO layers, the electrical characterization was conducted to investigate the transport properties of partially reduced GO layers obtained upon thermal treatment at 120 °C for 1 min. As discussed before, this temperature is within the safe window, in which no major chemical and structural changes occur in GO layers and the only purpose behind such mild annealing is to remove excess moisture from the sample To further vary the carbon-to-oxygen ratio, we utilized controlled voltage sweeping across the two-terminal GO device, which resulted in the generation of heat in the GO layers due to Joule heating. The Joule heating-induced reduction process not only modulated the conductivity of the device but also resulted in the observation of a unique NDR peak after a certain carbon-to-oxygen ratio is attained in the device. Fig. 3(a) illustrates the schematic of the measured device with the inset images showing the removal of oxygen functional groups as a result of Joule heating process. For the accurate Joule heating process, the voltage sweep (around 0~4 V) was repeated for 1^st^ ~9^th^ sweeps, as shown in Fig. 3(b). During the subsequent voltage sweeps, the current was gradually increased with the observation of the NDR behavior. Figure 3(c,d) show current-voltage (I–V) curves where controlled multiple sweeps were performed in ambient conditions. As shown in Fig. 3(c), the device exhibited a current of the order of few nano-amperes (nA) in the first sweep, whereas, the current increases by an order of magnitude with the subsequent sweep. This increase in current during voltage sweeping could be attributed to the Joule heating-induced reduction of the oxygen functional groups; GO was mainly reduced in the current flowing area and possibly at contact regions, where the reduction of the metal-GO layer barrier could result in an enhanced injection efficiency, as shown in the inset of Fig. 3(c). This technique has been employed by various research groups not only to reduce the oxygen functional groups of GO but also to improve the contact adhesion and channel conductivity in graphene-based devices, in which the Joule heating generated during several sweeping cycles effectively removes any volatile organic impurities and also improves the contact quality (see Supplementary Information, Section E, for detailed measurement of the Joule heating effect on GO devices). Interestingly, in the third voltage sweep, a peculiar and prominent NDR peak was observed along with an increase in current by an order of magnitude. The observed peak clearly show the characteristics of a NDR signal such as peak point, valley point, and negative differential resistance (${r}_{diff}=\frac{{\rm{\Delta }}v}{{\rm{\Delta }}i} < 0$) (Fig. 3(c,d)); this NDR peak maintained its repeatability even after 14 subsequent sweeps (see Supplementary Information, Section F, for further voltage sweep across the GO device). Although the subsequent sweeps maintained the NDR peak even at progressively increasing channel currents, the peak itself shifted to higher voltages. Notably, this continuous increase in peak current can be explained by the progressive Joule heating and additional removal of residual oxygen functional groups with every sweeping cycle. This observation of the NDR peak under optimum reducing conditions in GO devices cannot be explained along the lines of conventional NDR phenomena observed in solid-state devices like Esaki diodes and double quantum well heterostructures. In these conventional devices, concepts like interband tunneling and resonant/non-resonant electronic tunneling can satisfactorily explain the NDR behavior; however, a similar concept cannot describe the present scenario, where the band gap and the density of localized electronic states across the source and drain electrodes can vary, owing to the non-homogeneous distribution of various functional groups in the GO layers.

Owing to the constraint of availability of *micro* XPS to explore the variation in the fractions of various functional groups in the channel during repeated sweeping, ab initio NEGF simulation was performed for various coverage ratios of oxygen-based functional groups to assess the possible reasons behind the observation of NDR in the GO devices. An identical peak was obtained for a particular coverage ratio, which could be correlated with the condition in the experimental device at which the peak started to appear (see Supplementary Information, Section G, for detailed information on device simulations and comparison with experimental data).

**Figure 4:**
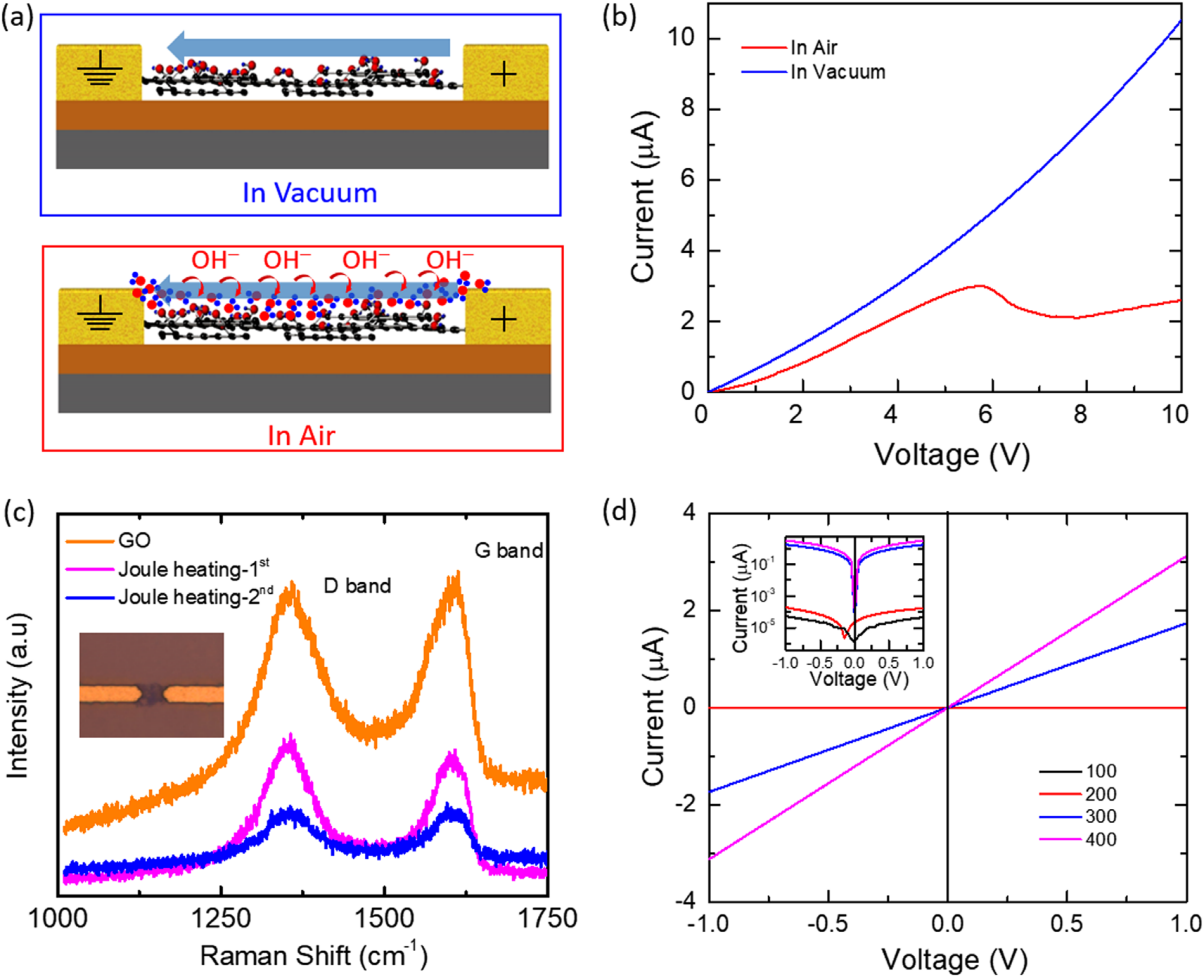
NDR mechanism in reduced graphene oxide device. (**a**) Schematic illustration of the device under measurement, where the upper image shows measurement under vacuum and the lower one in the ambient environment and the blue arrows in both schematics indicate current direction. Electric charge in the channel lowers the activation energy of electrochemical reactions, involving thin adsorbed water layer, which results in the chemisorption of the dissociated OH^−^ ions on the GO surface, as shown by curved red arrows. (**b**) I-V characteristics showing measurement in the ambient and vacuum environment. (**c**) Low power Raman spectra of GO and Joule heated GO, where the measurement was carried out twice to record the reduction in consecutive sweeps at the same spot. The inset shows a microscopic image of the sample after two electrical sweeps. (**d**) I-V characteristics of the GO device annealed from 100 ^o^C to 400 ^o^C with the inset figure showing the same data in semi-log scale.

However, such simulations under ideal transport conditions could be misleading due to high defect densities and non-ballistic carrier transport (variable range hopping) in rGO devices^41–43^. Although Joule heating-induced reduction process accounts for the irreversible increase in the device current, the exact reason behind the observation of NDR peak is still missing. To verify the origin of the observed NDR peaks, electrical measurements were carried out in the vacuum, after the occurrence of NDR peak in the ambient condition. As seen in Fig. 4(b), the I-V curve in the vacuum do not show NDR peak which indicates that the appearance of NDR peak after a certain threshold voltage is related to the ambient conditions. In the previous studies, the ambient conditions have found to influence the charge transport mechanisms through electrochemical reactions in the ambient environment and similar NDR peaks have been observed in I-V characteristics of ZnO coated peptide nanotubes and DNA thin films where it is activated by the dissociation of water molecules. (when the applied electric field reaches its electrochemical redox potential)^44,45^. In these studies, the NDR peak in electrical conductivity is attributed to protonic (H^+^ ions) conduction which is based on electrolysis of adsorbed water (Grotthuss mechanism) at the electrodes. The enhancement in conductivity is quickly limited by the depletion and slow diffusion of an adsorbed layer of water near the electrodes, thus resulting in NDR behavior. However, such electrochemical reactions at the electrodes in GO devices are further complicated by the electric field and charge induced redox process as the activation energy of various oxygen functional groups in GO can be manipulated by the applied electric field at electrodes and GO channel as well^46,47^. Such reduction processes can be reversible at lower voltage and vice-versa and are facilitated by the charge injection into GO which weakens the bonds of negatively charged oxygen functional groups covalently attached to the underlying carbon frame, as follows^48^;

1 $${{\rm{H}}}_{{\rm{2}}}{\rm{O}}\to {{\rm{4H}}}^{+}+{{\rm{4e}}}^{-}+{{\rm{O}}}_{{\rm{2}}}\,\,\,({\rm{oxidation}}\,{\rm{of}}\,{\rm{adsorbed}}\,{\rm{water}})$$

2 $${\rm{GO}}+{{\rm{2H}}}^{+}{+\mathrm{2e}}^{-}\to {\rm{rGO}}+{{\rm{H}}}_{{\rm{2}}}{\rm{O}}\,({\rm{reduction}}\,{\rm{of}}\,{\rm{GO}})$$

As GO belongs to the family of 2D materials, having a large surface to volume ratio, along with hydrophilic surface due to oxygen based functional groups, another mechanism behind the observation of NDR can be explained by the electrochemical reaction of the ambient O_2_ and a thin layer of water adsorbed at the GO surface. The current flowing in the underlying channel facilitates the reaction by lowering the activation and binding energy of oxygen, resulting in the following electrochemical reaction^49^;

3 $${{\rm{O}}}_{{\rm{2}}}+{{\rm{2H}}}_{{\rm{2}}}{\rm{O}}+{{\rm{4e}}}^{-}\to 4{{\rm{OH}}}^{-}$$

This electrochemical process which generates OH^−^ ions results in p-type doping effect upon the chemisorption of the OH^−^ ions on the GO channel and leads to a rapid increase in the channel current, as illustrated in the schematic of Fig. 4(a). This rapid increase in the channel current is swiftly limited by the depletion and slow diffusion of the thin adsorbed layer of water on the GO channel, thus resulting in the NDR peak^44,49^. Unlike in the previous studies, where repeated observation of NDR was missing due to the limited rate of water diffusion, GO device do not show such relaxation and NDR can be observed in subsequent sweeps as well. This can be explained by the large surface to volume ratio and high density of hydrophilic functional groups which can facilitates quick adsorption of water from the ambient environment^46,48^. However, the rate of water depletion and adsorption depend upon various factors including ambient humidity, temperature and hydrophilic behavior of GO, which decreases with the reduction of GO.

It may be noted that, though the observed NDR in GO devices is related to the ambient conditions, an optimum conductivity of GO channel is necessary to observe NDR via doping or reversible reduction effect of the electrochemical induced process. If the conductivity or carbon-to-oxygen ratio in the GO channel is less than the optimum value then NDR could not be observed due to incomplete channel formation (insulating regime, GO) whereas if the conductivity is higher or GO is highly reduced then the effect of electrochemical doping becomes negligible (semi metallic regime, fully reduced GO), as can also be seen from the linear I-V curve for thermally (>300 ^o^C) reduced GO, Fig. 4(d))^43^. Therefore, NDR can only be observed in the narrow conductivity regime (semiconducting regime, partially reduced GO), which can be controlled by the hybrid annealing method as demonstrated in this study. Figure 4(c) illustrate the Joule heating induced reduction of GO channel which is irreversible unlike low voltage induced reversible reduction, which also becomes irreversible, once the channel (reduced GO) formation is completed by the combination of both the electric field and current induced reduction (Joule heating) processes^46,47^. (see Supporting Information, Section B, for detailed information on thermal and Joule heating based reduction of GO). Finally, the NDR observed in the lateral GO devices is completely different from the noise and random peaks usually observed in the I-V characteristics of devices based on GO or other oxides, as the NDR peaks in this study are repeatable and follow a definite trend, unlike peaks due to random charge trapping and emission at high density of defect sites in GO or other oxides^49^.

In summary, the results demonstrate the NDR behavior in GO layers by adopting the hybrid thermal and Joule heating reduction processes. The NDR behavior was correlated with optical and chemical characterization, which shows good agreement with the experimental results. The observed NDR peak in the optimized devices is attributed to the charge-activated electrochemical reactions at GO and ambient interface, which doped the GO channel until the adsorbed thin layer of water is not depleted. As GO is a strong candidate for several device applications, these findings can be applied to study charge induced chemical reactions and to fabricate GO based chemical and pH sensors.

## Methods

### Synthesis of graphene oxide solution

The GO nanostructures were synthesized using a modified Hummer’s method^26^. First, 4 g of graphite flakes was added to a round-bottom flask (250 ml) containing 120 ml of H_2_SO_4_; the solution was then stirred for 1 h. During stirring, KMnO_4_ aqueous solution was added to the mixture every 20 min. The mixture was slowly heated to 40 °C, and the temperature was maintained for 5 h to oxidize the graphite. Subsequently, 150 ml of deionized (DI) water was added to the mixture, stirring for 30 min while 17 ml of H_2_O_2_ solution was added. The mixture was subsequently maintained at 40 °C for 24 h and then centrifuged. The resultant mixture was enclosed in a dialysis tube and washed with ultrapure DI water several times to obtain a pH level of 5. The concentration of the GO nanostructure solution was ~1 mg/ml.

### Characterization with Raman and XPS spectroscopies

To confirm the quality (chemical bonding, electrical and structural properties) of GO layers, XPS (ESCA 2000, VG Microtech, UK) was performed using twin anode X-ray sources K_α_ (1486.6 eV)/Mg K_α_ (1253.6 eV) in a vacuum of 10^−9^ Torr, whereas Raman spectra were obtained at room temperature using a 532 nm laser.

### Device fabrication

For device fabrication, pre-patterned SiO_2_/Si wafers having Ti/Au electrodes (gap of 2–11 μm) were used. For GO assembly between the electrodes, 0.1 μl of GO solution was dropped onto the electrode area. After evaporation of the liquid, the sample was allowed to dry at 90 °C for 5 s, and then annealed at 120 °C for 10 s under ambient conditions. This was followed by repeated I–V sweeps to achieve controlled Joule heating-induced reduction of the GO layers.

### Device Simulations

ATK^®^ utilizes DFT in conjunction with NEGF formalism for computing electronic transport related quantities^50–52^. In ATK^®^, the DFT-NEGF parameters were selected as follows: electron temperature of 300 K, mesh cut-off energy of 200 Ry, Monkhorst-Pack sampling of (1,1,100) for the Brillouin zone, and local density approximation (LDA) as the exchange-correlation functional^53^. The basis set was chosen as double-zeta polarized, and Dirichlet boundary conditions were used for the fast Fourier transform (FFT) grids.

## Electronic supplementary material

Supplementary Information
