# Supplementary material for: Observation of negative differential resistance in mesoscopic graphene oxide devices
Source: Sci Rep. 2018 May 8;8:7144. doi: 10.1038/s41598-018-22355-0 (PMC5940784; doi:10.1038/s41598-018-22355-0)
Supplement: Supplementary file 1 — Supplementary Information [file 41598_2018_22355_MOESM1_ESM.docx]

Observation of negative differential resistance in mesoscopic graphene oxide devices

Servin Rathi^†1^, Inyeal Lee^†1^, Moonshik Kang^1,2^, Dongsuk Lim^1^, Yoontae Lee^1^, Serhan Yamacli^3^, Han-Ik Joh^4^, Seongsu Kim^5^, Sang-Woo Kim^5^, Sun Jin Yun^6^, Sukwon Choi^7^, and Gil-Ho Kim^1*^

*^1^Samsung-SKKU Graphene Center, Sungkyunkwan Advanced Institute of Nanotechnology (SAINT) and School of Electronics and Electrical Engineering, Sungkyunkwan University, Suwon 16419, Korea*

*^2^Manufacturing Engineering Team, Memory Division, Samsung Electronics Co., Hwasung 18396, Korea*

*^3^Department of Electrical-Electronics Engineering, Nuh Naci Yazgan University, 38090 Kayseri, Turkey*

*^4^Department of Energy Engineering, Konkuk University, 120 Neungdong-ro, Gwangjin-gu, Seoul 05029, South Korea*

*^5^School of Advanced Materials Science and Engineering, SKKU Advanced Institute of Nanotechnology (SAINT), Center for Human Interface Nanotechnology (HINT), and IBS Center for Integrated Nanostructure Physics, Sungkyunkwan University, Suwon 16419, Korea*

*^6^ICT Components and Materials Technology Research Division, Electronics and Telecommunications Research Institute, Daejeon 34129, Korea*

*^7^Department of Mechanical and Nuclear Engineering, The Pennsylvania State University, University Park, PA 16802, USA*

*corresponding author Gil-Ho Kim, E-mail: ghkim@skku.edu

^†^ These authors contributed equally to this work

**Main Contents**

1. **AFM Images of etched GO two-terminal device**
2. **Low power Raman spectra of thermal and Joule heating induced reduced GO**
3. **O1s XPS spectra for GO and thermal reduced GO**
4. **Electrical characteristics of thermally annealed I-V for different GO concentration**
5. **Optical imaging of GO reduction via Joule heating**
6. **Repeatability of NDR peaks with subsequent voltage sweeps in the GO device**
7. **ab initio NEGF simulations**
8. **References**
9. **AFM Images of etched GO two-terminal device**

To estimate the thickness of the deposited graphene oxide (GO) layers in between the electrodes, Atomic force microscopy (AFM) technique was employed. For this, the deposited GO layer on the pre-patterned electrodes was etched using typical photolithography technique and oxygen plasma etching, resulting in a well-defined GO channel. Fig. S1 shows AFM profile of various GO samples with a line profile across the edge of the sample (c) with an average height of 28 nm. Through various AFM 3D and plane profiles, we analyzed the thickness of the deposited GO layers and were found out to be non-uniform, as expected from the deposition method using solution based GO. Also, the residues seen in the profile AFM image are organic residue, which remained after the photolithography processing.


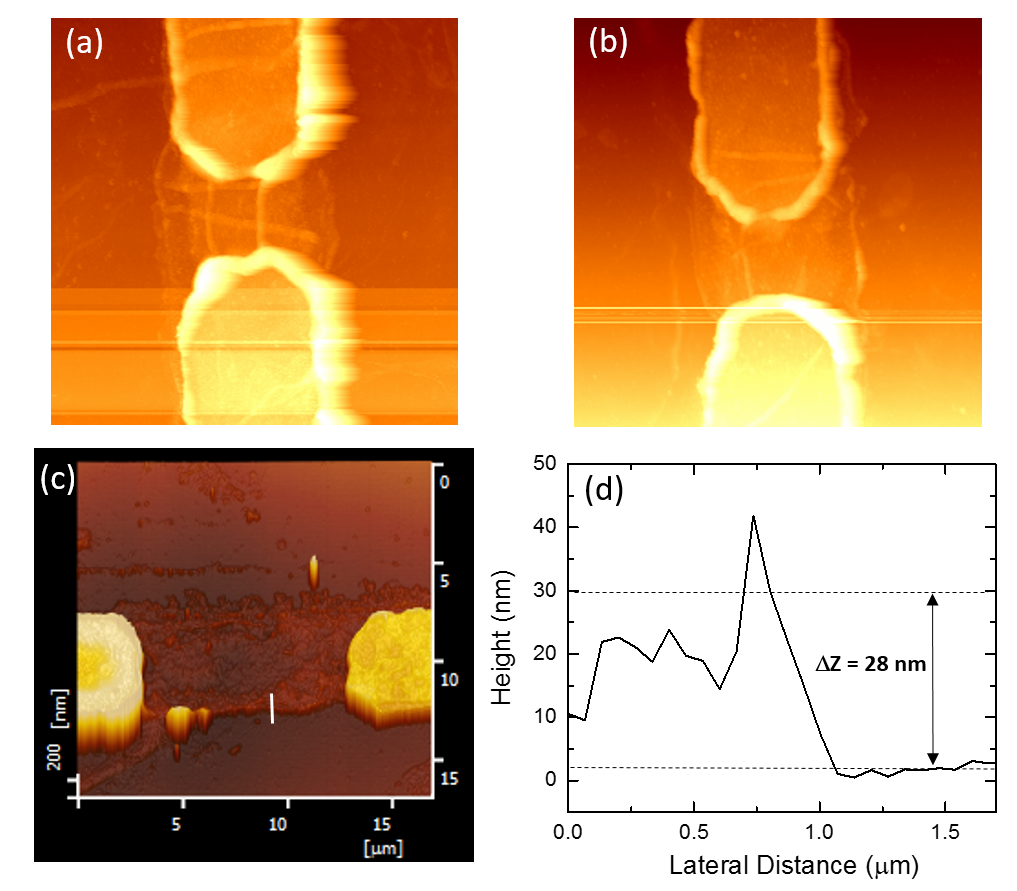


**Fig. S1. AFM profile of various GO samples.** (a, b) AFM plane profiles, and (c) 3D AFM image of the etched GO channel device. (d) AFM line profile across the edge of GO channel along the white line shows the thickness of 28 nm.

1. **Low power Raman spectra of thermal and Joule heating induced reduced GO**

A low-power micro Raman measurement (50 μW, 20 sec exposure using 514 nm laser source) to study the changes in GO devices after Joule heating effects. Raman measurement of pristine GO device and on both thermal reduced GO and Joule heating induced reduced GO i.e. after the observation of NDR effect were carried out. For this, the laser power induced reduction of GO samples was minimized before actual measurement by optimizing the laser exposure time for low power laser beam.


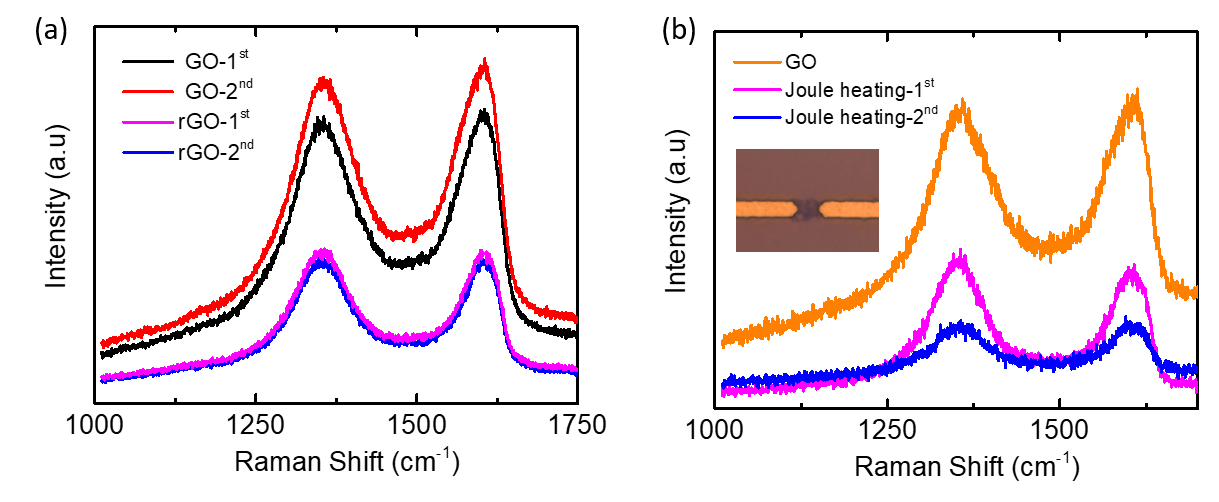


**Fig. S2**. a) Raman spectra of GO and thermally reduced GO samples. Raman spectra were recorded twice at the same spot so as to confirm the thermal reducing effects of the laser spot. b) Raman spectra of GO and Joule heated GO where the measurement was carried out twice to record the reduction in consecutive sweeps at the same spot. The inset shows a microscopic image of the sample after two electrical sweeps. *(Note: this figure is repeated from the main text to illustrate and compare the Raman spectra of thermal and Joule heating based rGO)*

As can be seen from the figure S2 that both thermally reduced GO and Joule heating reduced GO results in the fall of spectra intensity and increase in the intensity ratio of D and G peaks, (I_D_/I_G_ <1 in GO and >1 in reduced GO) which clearly indicates reduction in Joule heating process, similar to that of thermally reduced GO.

Further, the Joule heating based reduction has been reported by several studies. In one study,^1^ Joule heating induced temperature of 2750 K was achieved by the current of mA order whereas in another study,^2^ a temperature of 600 ^o^C was achieved by Joule heating in ambient temperature of 76 K. As the current order in partially reduced GO depends on various factors like bias voltage, the degree of reduction, sample size, length, and width, so the higher order current reflects highly reduced GO as a results of very high-temperature annealing induced by Joule heating. However, in the present study, only a partial annealing is the main aim and a temperature in the range of 200 ^o^C– 400 ^o^C can be achieved by nA or μA current in the device. Further, fully reduced GO devices of L= 10 μm and width = 4 μm shows a current of the order of 50 μA., which can be increased to mA by reducing the device length or increasing its width.^3^ Therefore, in the present study, Joule heating induced reduction through controlled sweep is considered to be partial and do not fully reduced the GO to rGO as in the case of high temperature reduced GO.

1. **O1s XPS spectra for GO and thermal reduced GO**


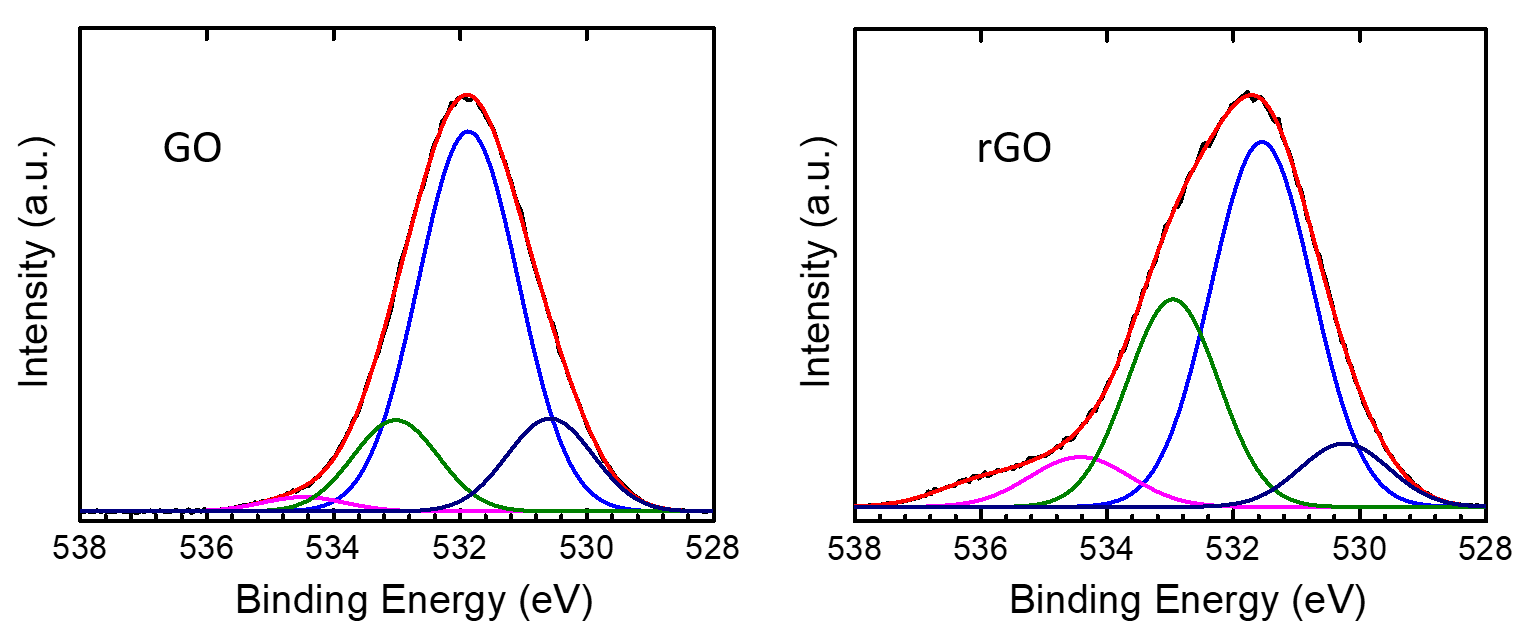


**Fig. S3.** O1s XPS spectra of GO and rGO annealed at 400 ^o^C.

As can be seen from the Fig. S3 that the reduction of GO to rGO affect the peaks distributions at various binding energy of 530.5 eV, 532 eV, 533.2 eV and 534.5 eV. These peaks represent various oxygen bonding via single and double bonds to aromatic or aliphatic carbon (C=O, C-O, etc.).^4,5^ The thermal reduced GO results in shifting of XPS spectra to higher energy side and transformation of single peak data in GO to a dual one in rGO. A decrease in the peak at 530.5 eV also indicates the loss of oxygen during reduction treatment which agrees with C1s peak spectra as well.

1. **Electrical characteristics of thermally annealed I-V for different GO concentration**

As GO can be reduced to rGO (reduced GO) via various methods including chemical, optical, thermal and Joule heating. Therefore, in order to confirm the role of thermal annealing in the observed NDR behavior which was achieved through an optimized combination of thermal and Joule heating, some exclusively thermal annealed devices were analyzed for various concentration


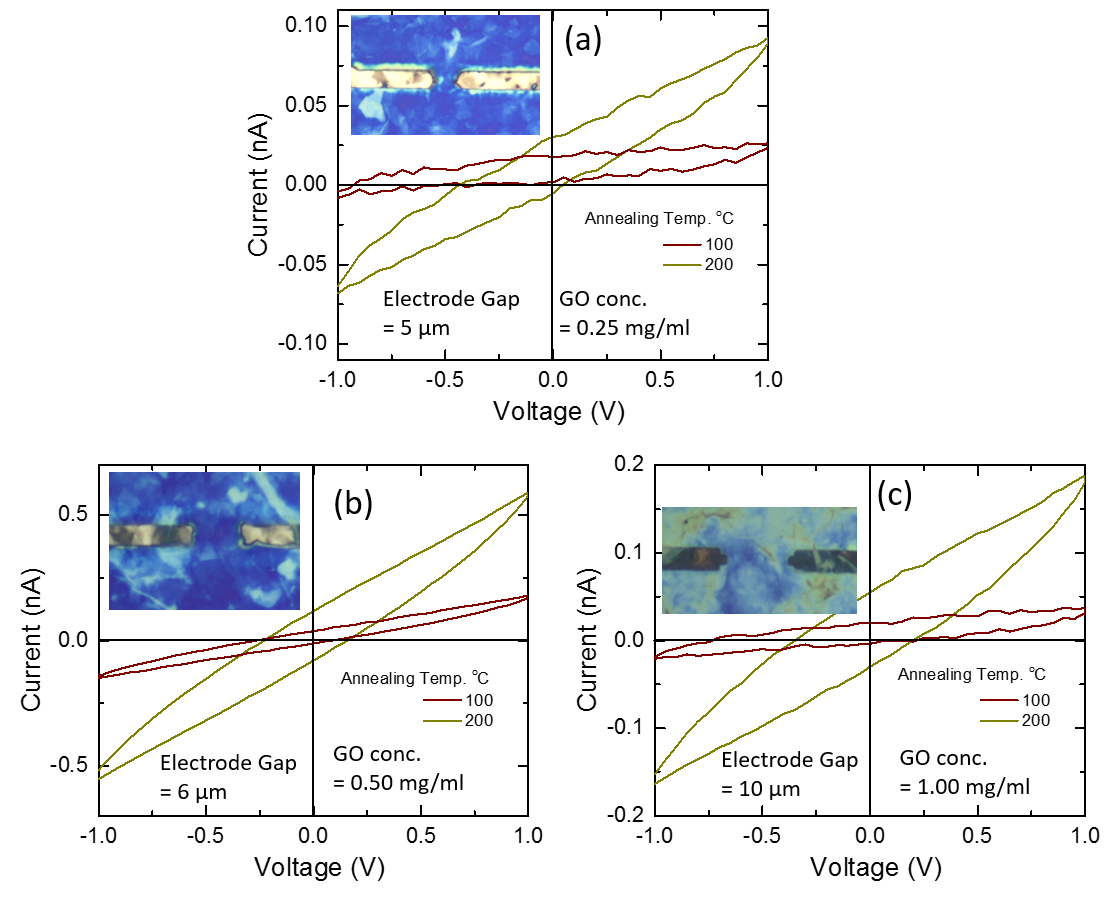


**Fig. S4**. **Thermal annealing and electrical measurement of the GO devices for various GO concentration and annealing temperature.** The I-V characteristics of the GO device annealed at 100 ^o^C and 200 ^o^C for (a) 0.25, (b) 0.50, (c) 1.00 mg/ml concentration of GO solution. The inset in the Fig. (a-c) show the optical microscopic image of the fabricated devices.

and annealing temperature conditions. For GO concentration of 0.25, 0.50 and 1.00 mg/ml, several devices with variable electrode gaps ranging from 1.8 µm up to 12 µm were fabricated and subsequently annealed at a temperature varying from 100 to 400 ^o^C. From Fig. S4, it can be seen that for lower temperature 100-200 ^o^C annealing, the oxygen functional groups were not completely reduced, which resulted in low current in the devices.


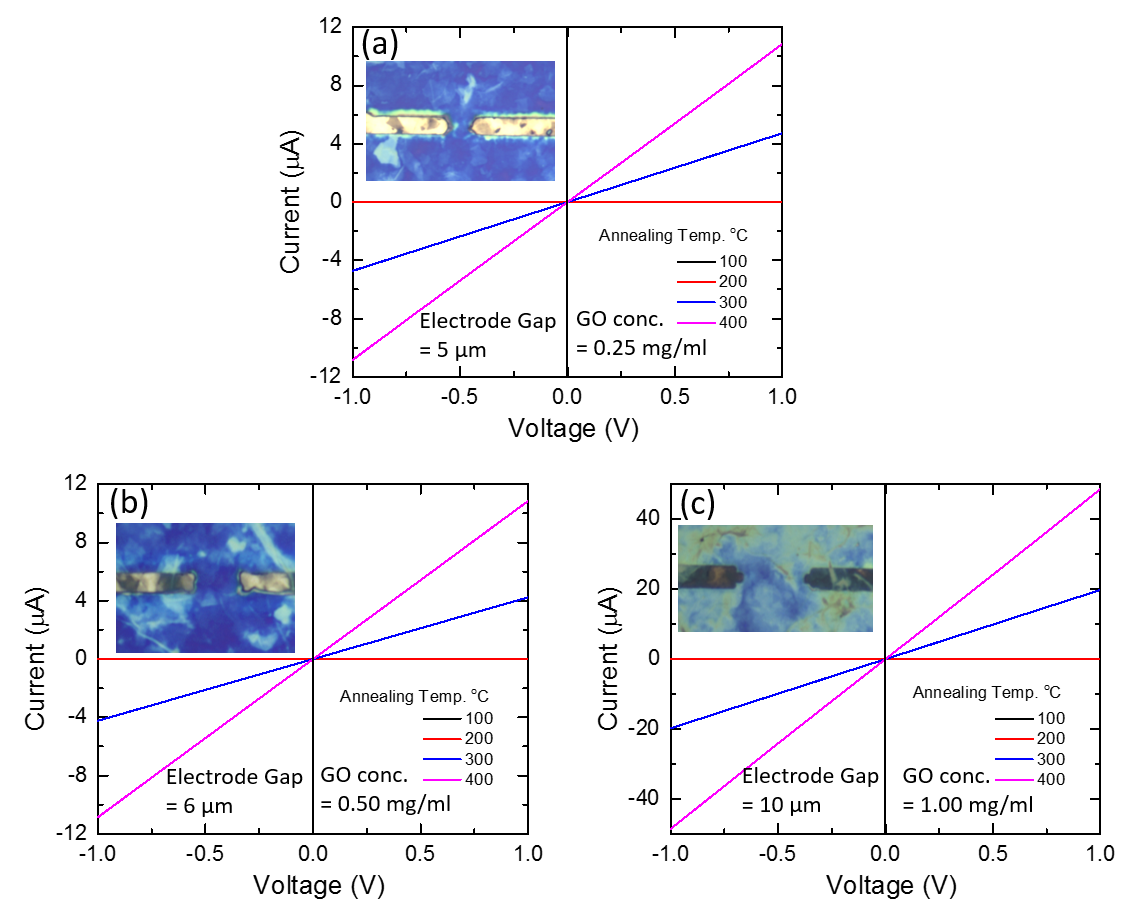


**Fig. S5**. **Thermal annealing and electrical measurement of the GO devices for various GO concentration and annealing temperature.** The I-V characteristics of the GO device annealed from 100 ^o^C to 400 ^o^C for (a) 0.25, (b) 0.50, (c) 1.00 mg/ml concentration of GO solution. The inset in the Fig. (a-c) show the optical microscopic image of the fabricated devices.

However, for 300 and 400 ^o^C annealing temperature, the current in the device jumped from less than nA to the order of µA, see Fig. S5. This current jump coincides with no hysteresis and matches well with the observed XPS data (in the main text, Fig. 2), which shows an enormous reduction in the oxygen functional groups at 300 and 400 ^o^C. Although, a steep current jump was observed for all the devices under test, we have not observed NDR behavior in these devices, which illustrates that only thermal annealing-based reduction process is not suitable to observe the NDR effect and an optimum control of carbon-to-oxygen ratio is required.

1. **Optical imaging of GO reduction via Joule heating**

The facile thermal annealing process and subsequent XPS and I-V measurement confirm the reduction of the GO, whereas to demonstrate the Joule heating based reduction of GO on the application of the applied electric field, we have followed the optical imaging contrast technique based on the variation in GO transmittance while it undergoes reduction. For this measurement, the devices were coated with PMMA for higher optical contrast as compared to without coated devices, with no observable effect on the I-V characteristics. Fig. S6 shows the I-V characteristics under various sweep cycles. It can be seen that as the device undergoes repeated voltage sweeping, a darker region is developed in between the region connected the electrodes. On subsequent sweeping, this contrast region expands beyond the channel region as well. This has been linked to the electric field induced reduction of the GO and has also been studied in detail elsewhere^6,7^.

In addition to the current induced Joule heating, GO can also be reduced by the electric field induced electrochemical reduction of oxygen ions based species and electron injection based weakening of negatively charged oxygen bonds.^6^ This is also evident from the microscopic images in Fig. S6 where the reduced region continues to expand beyond the current flowing region between the electrodes. This region continues to expand in the subsequent sweeps 4-5, in which the reduction can be clearly seen in the large vicinity area around electrodes, which is beyond the immediate channel region. Such kind of field-induced reduction can be reversed^6^, however, after the channel formation, the current in channel results in Joule heating based irreversible reduction of the main GO channel.


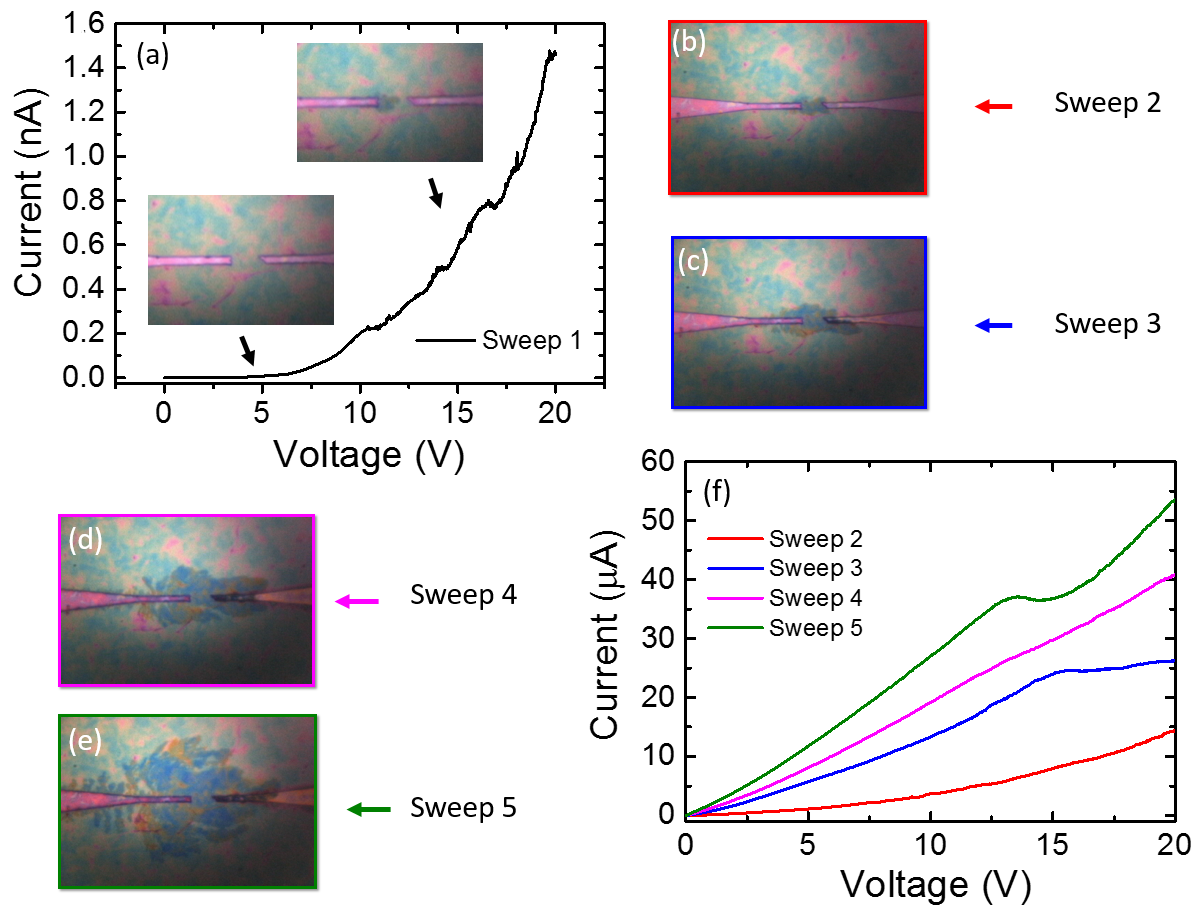


**Fig. S6**. **Optical images of the GO layers as it goes through Joule heating induced reduction.** (a) First I-V sweep and the inset showing the optical image of the device at the beginning and middle of the sweep (b-e) Optical Images of the subsequent sweep from 2 to 5 and (f) showing the I-V characteristics of the respective sweep.

Further, the abnormal missing of NDR peak in sweep 4 (Fig. S6), after appearing in sweep 3, could be possibly due to the formation of highly reduced or conductive filament channel in the reduction process, thus resulting in almost linear curve or due to a brief depletion of thin adsorbed layer of water on the GO surface. However, in the subsequent sweep, this narrow filament path or channel is no longer the least resistive path possibly due to electron migration of charges under the influence of applied electric field resulting in oxidation and/or the formation of other reduced channels at the same time with appropriate C-O ratio thus the recurrence of NDR curve. As the GO sample is multilayered therefore a complex mechanism where electric field induced electrochemical reduction and oxidation and migration of oxygen-based hydroxyl, carboxyl species can also lead to such abnormal results. Further, no such abnormality was observed in the measurement with other samples, as shown in Fig. S8.

1. **Repeatability of NDR peaks with subsequent voltage sweeps in the GO device**

The NDR peak as observed in the main text maintained its repeatability even after 5 subsequent sweeps as shown in Fig. S7. As seen from the figures that with each subsequent sweep, the channel current increases and the peak shifted to higher voltages. The progressive Joule heating and additional removal of residual oxygen functional groups can explain the continuous increase in the channel current with every subsequent voltage sweep. Fig. S8 shows results from another sample when exposed to similar controlled sweeping conditions.


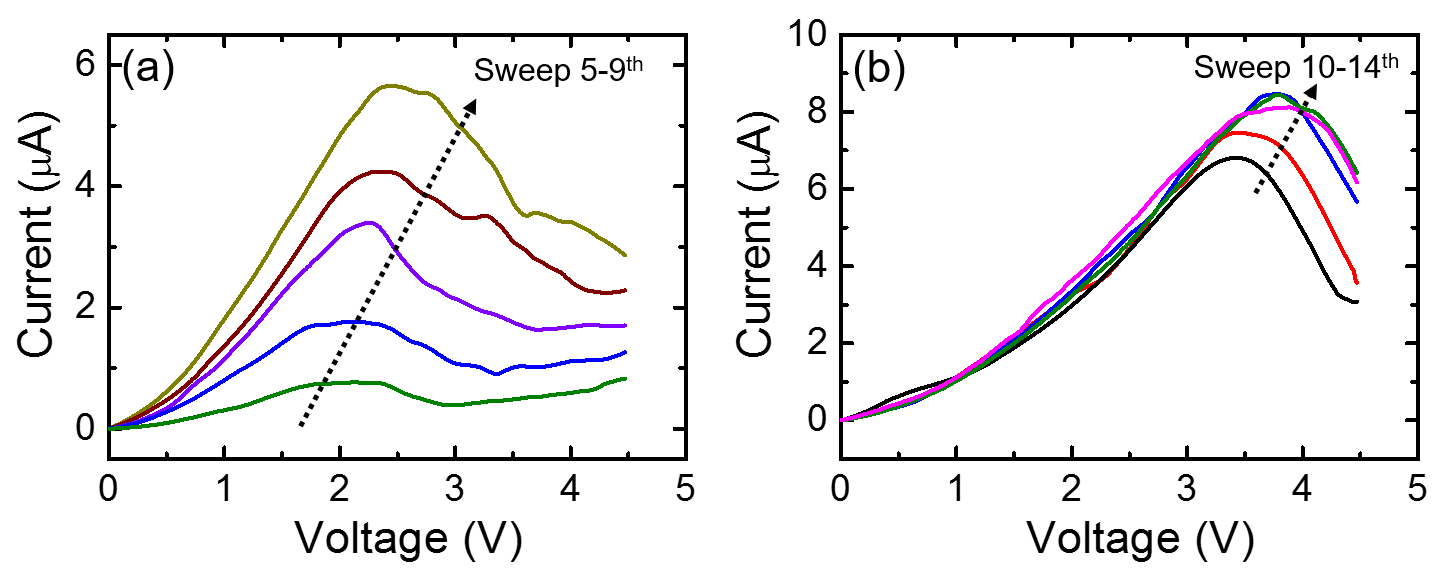


**Fig. S7**. I–V sweeps from (a) 5 to 9^th^ sweep and (b) 10 to14^th^ sweep, with all the sweeps showing the NDR peaks.


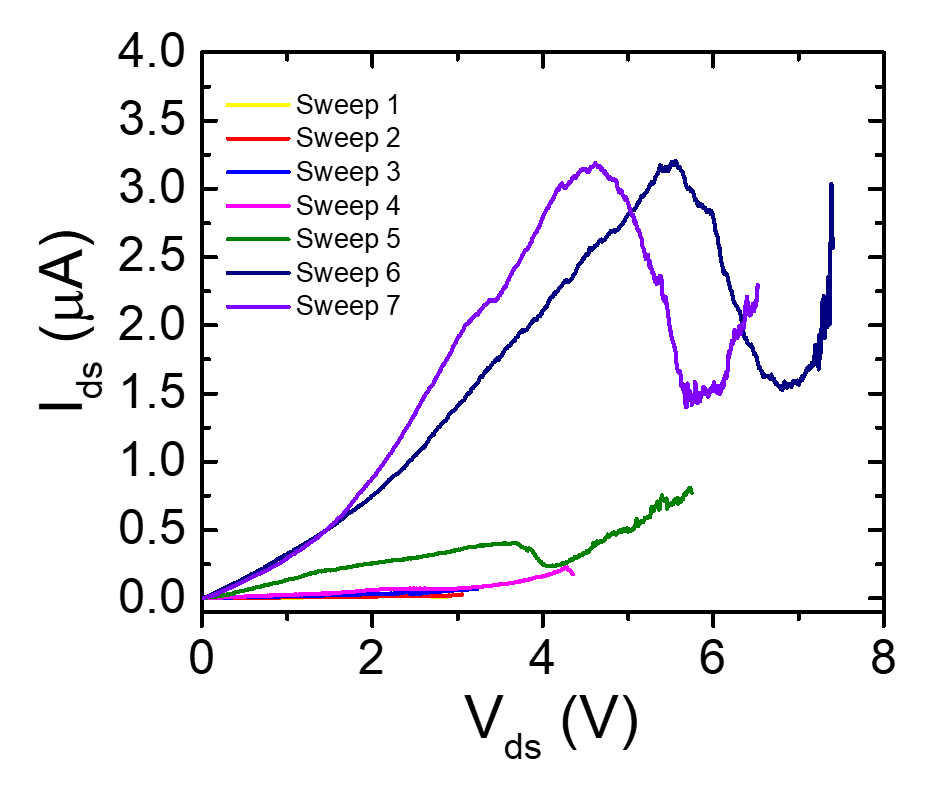


**Fig. S8**. I-V curve for repeated voltage sweep showing NDR peaks after few initial controlled sweeps.

**G. ab initio NEGF simulations**

Various nanostructures including nanowire, molecular, and GNR based devices can be accurately modeled using ab initio simulation methods.^8–10^ There are several self-consistent methods and simulation tools in the literature, providing a differing level of accuracies. In this study, Atomistix Toolkit (ATK^®^) from Quantumwise A/S^11^ was used for simulating the GO structure decorated by different coverage ratios of functional groups. This simulation package was chosen as it provides I–V characteristics with proven accuracy. In ATK^®^, the I–V characteristics are calculated using Landauer’s formula:^12^

$I=\frac{e}{h}\int_{-\infty}^{\infty} T(E)\left[ f\left( E-\mu_{L} \right)-f(E-\mu_{R}) \right]dE$ (1)

where *h* is Planck’s constant, *e* is the elementary charge, *E* is the energy, *µ_L_* and *µ_R_* are the electrochemical potentials of the left and right electrodes, respectively, *f(E-µ_L_)* and
*f(E-µ_R_)* are the Fermi-Dirac probability distribution functions of the electrodes, and *T(E)* is the
energy-dependent transmission function calculated using DFT-NEGF approach in ATK^®^; 101 voltage points between 0 and 4 V were used for the computation of the characteristics. First, the geometry of the GO structure is optimized in ATK^®^, and then the actual I–V curve calculation is performed using Pulay Mixer algorithm with a damping factor of 0.5. The GO structure for simulation was considered as mono-layer containing oxygen functional groups,


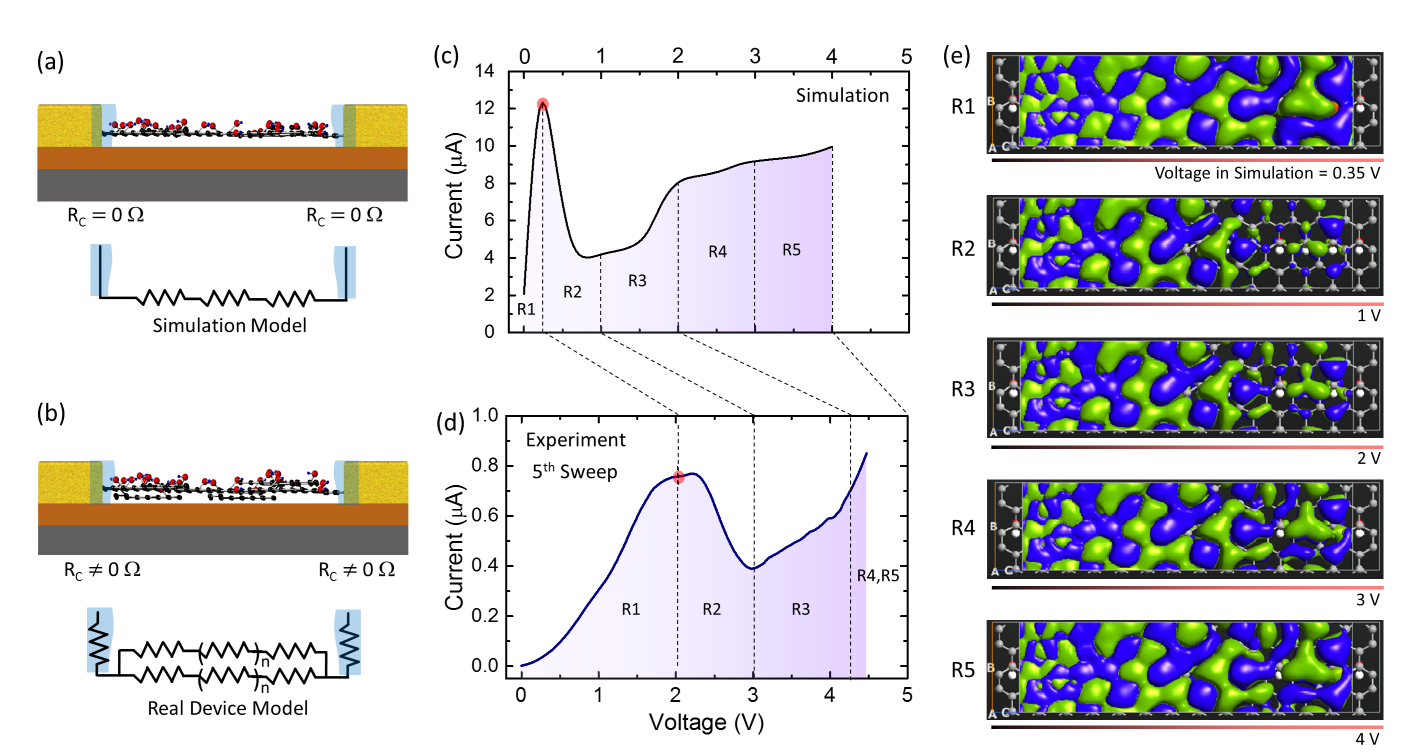


**Fig. S9.** Ab initio analysis of electrical transport in the graphene oxide device. Schematics of the Ohmic consideration in the (a) simulated and (b) fabricated devices. I–V characteristics of the (c) simulated and (d) real GO device. (e) Transmission eigenstates of the simulated rGO structure for the applied voltages from 0.35V to 4 V. Although the curves look similar, but the NDR process is completely different in both the devices.

and the contact resistance at GO-metal interface was neglected as shown in Fig. S9(a-b). The simulated I-V plot shows a clear NDR characteristic, and the curve shape is almost similar to that of 5^th^ sweep I-V curve of the real device, as shown in Fig. S9(c) and (d).

Further, the simulation analysis shows that the observation of NDR in the optimized device could be linked to the dependence of the transmission spectrum on the applied voltage. As the conductance is dependent on the voltage-dependent transmission spectra and this dependence is related to the carbon-to-oxygen ratio in partially reduced GO which results in the appearance of NDR peak at an optimized value.^13^ This NDR behavior can be explained from the dependence of transmission eigenstates and their localization and delocalization, which represent the low and high transmission probability of electrons through the structure, respectively, as shown in Fig. S9(e). This dependence of transmission eigenstates and their localization degree on the applied voltage satisfactorily explains the NDR behavior in the simulated device. Previous study has shown that the NDR behavior at optimized oxygen functional groups can give way to linear I-V characteristics for pristine or very high carbon-to-oxygen ratios.^13^

However, it may be noted that though there are many apparent similarities in the simulated and experimental results, any real correlation between them is elusive due to the fact that the simulation study is based on Landauer’s formula which does not account for any inelastic carrier transport in GO devices. Therefore, only ideal carrier transport in pristine graphene devices under various oxygen coverage can be correlated to the simulated results. But, as the liquid exfoliated GO has a high density of traps and defects states and previous studies have shown that the conduction mechanism in GO devices is via variable range hopping,^14^, therefore, any similarities between the above simulated and experimental data cannot be compared as such because NDR is observed due to an altogether different mechanism in the respective devices.

**H. References**

1. Chen, Y. *et al.* Reduced graphene oxide films with ultrahigh conductivity as Li-ion battery current collectors. *Nano Lett.* **16,** 3616–3623 (2016).

2. Moser, J., Barreiro, A. & Bachtold, A. Current-induced cleaning of graphene. *Appl. Phys. Lett.* **91,** 163513 (2007).

3. Wang, J. *et al.* Alternating Current Dielectrophoresis Optimization of Pt-Decorated Graphene Oxide Nanostructures for Proficient Hydrogen Gas Sensor. *ACS Appl. Mater. Interfaces* **7,** 13768–13775 (2015).

4. Tang, L. *et al.* Bottom-up synthesis of large-scale graphene oxide nanosheets. *J. Mater. Chem.* **22,** 5676 (2012).

5. Ganguly, A., Sharma, S., Papakonstantinou, P. & Hamilton, J. Probing the Thermal Deoxygenation of Graphene Oxide Using High-Resolution In Situ X-ray-Based Spectroscopies. *J. Phys. Chem. C* **115,** 17009–17019 (2011).

6. Ekiz, O. O., Ürel, M., Güner, H., Mizrak, A. K. & Dâna, A. Reversible electrical reduction and oxidation of graphene oxide. *ACS Nano* **5,** 2475–2482 (2011).

7. Mativetsky, J. M. *et al.* Graphene transistors via in situ voltage-induced reduction of graphene-oxide under ambient conditions. *J. Am. Chem. Soc.* **133,** 14320–14326 (2011).

8. Srivastava, A., Tyagi, N. & Ahuja, R. First-principles study of structural and electronic properties of gallium based nanowires. *Solid State Sci.* **23,** 35–41 (2013).

9. Min, Y. *et al.* Disconnect armchair carbon nanotube as rectifier predicted by first-principles study. *Comput. Mater. Sci.* **81,** 418–422 (2014).

10. Min, Y. *et al.* Bias changing molecule–lead couple and inducing low bias negative differential resistance for electrons acceptor predicted by first-principles study. *Phys. Lett. A* **379,** 2637–2640 (2015).

11. Atomistix Toolkit version (2014.3.): Quantumwise A/S. www.quantumwise.com.

12. Datta, S. *Quantum Transport: Atom to Transistor*. (Cambridge University Press, 2005). doi:10.1017/CBO9781139164313

13. Yamacli, S. Voltage-dependent electronic transport properties of reduced graphene oxide with various coverage ratios. *Nano-Micro Lett.* **7,** 42–50 (2014).

14. Joung, D. & Khondaker, S. I. Efros-Shklovskii variable-range hopping in reduced graphene oxide sheets of varying carbon sp2 fraction. *Phys. Rev. B - Condens. Matter Mater. Phys.* **86,** 1–8 (2012).
